# Preservation and Management of the Superficial Nasal Musculoaponeurotic System to Enhance Infratip Contour: A Technical Report

**DOI:** 10.7759/cureus.117772

**Published:** 2026-10-11

**Authors:** Alan Burgos, Ilein A Rivera Navarrete

**Affiliations:** 1 Rhinology and Facial Plastic Surgery, Private Practice, Mazatlán, MEX; 2 Otolaryngology-Head and Neck Surgery, Instituto Nacional de Rehabilitación, Mexico City, MEX

**Keywords:** infratip, nasal columella, nasal ligaments, nasal tip, rhinoplasty surgery, soon flap stitch

## Abstract

This technical report describes a surgical technique developed to manage the dead space between the osteocartilaginous framework and the superficial musculoaponeurotic system (SMAS) during open rhinoplasty. The technique utilizes the superficial orbicularis oris nasalis (SOON) muscle to preserve the superficial layer of the nasal SMAS within the columella, with the aim of maintaining columellar volume and anatomy while minimizing dead space formation. During an open rhinoplasty approach, the SOON muscle is preserved through subperichondrial dissection to create a vascularized flap (SOON flap). The flap is subsequently repositioned and secured using the SOON Flap Stitch, a suture technique developed by the author (IARN) to restore the preserved tissue over the nasal tip framework. The technique was performed in 226 consecutive primary open rhinoplasty procedures, with postoperative follow-up ranging from eight months to three years. The senior author (AB) observed reduced dead space and columellar retraction, as well as improved infratip volume, definition, and double-break contour. The SOON Flap Stitch represents a simple and reproducible technique for preserving and repositioning the superficial nasal SMAS, promoting its redraping and integration over the underlying osteocartilaginous framework while enhancing columellar and infratip contour in open rhinoplasty.

## Introduction

Rhinoplasty remains one of the most technically demanding procedures in facial plastic surgery. Variations in nasal anatomy, skin thickness, and wound healing significantly influence postoperative outcomes, making the quality of the soft-tissue envelope a critical determinant of surgical predictability. The skin-soft-tissue envelope consists of the skin and underlying soft tissues that cover the nasal osseocartilaginous framework formed by the nasal bones and cartilages. During rhinoplasty, surgical dissection separates these layers and may create a potential space between the soft-tissue envelope and the reconstructed framework, commonly referred to as dead space. Persistent dead space may interfere with predictable soft-tissue redraping and adaptation during healing, potentially contributing to fibrosis and postoperative contour irregularities. Therefore, controlled repositioning of the soft-tissue envelope over the reconstructed nasal framework is an important component of structural rhinoplasty [1].

Throughout the evolution of rhinoplasty, numerous authors have described supporting structures, commonly referred to as nasal retaining ligaments, that connect the osseocartilaginous framework to the superficial musculoaponeurotic system (SMAS). Preservation or repositioning of these structures has been proposed to improve postoperative nasal support and soft-tissue adaptation [2-5]. However, the terminology and anatomical interpretation of these structures remain controversial, with multiple authors describing similar anatomical findings under different names [6-8].

Within the current ligament-based nomenclature, the superficial fibromuscular layer is considered the superficial portion of the SMAS. More recently, Neves et al. introduced the concept of the Fusion Sling, recognizing that the intercrural compartment, defined as the soft-tissue space between the medial crura of the lower lateral cartilages, is occupied by the superficial orbicularis oris nasalis (SOON) muscle [6]. Building upon this contemporary understanding of nasal suspensory anatomy, the SOON Flap Stitch technique was developed to preserve and reposition this superficial SMAS layer over the columellar framework, with the aim of restoring anatomical continuity, minimizing dead space, and maintaining infratip and columellar soft-tissue volume.

Anatomical background

The understanding of nasal support mechanisms has evolved considerably over the past several decades. Early cadaveric studies sought to identify discrete anatomical structures responsible for maintaining nasal tip position and projection, leading to the widespread description of so-called nasal ligaments. Nevertheless, their anatomical definition, nomenclature, and surgical relevance have remained subjects of ongoing debate.

One of the landmark contributions was the doctoral thesis by Bruintjes et al. in 1996, which described horizontal and vertical fibrous condensations within the intercrural and scroll regions, derived from the embryologic fusion of the medial and lateral nasal processes [2]. Histologic analysis demonstrated that these condensations represent bilateral fusion connective tissue rather than an independent midline structure. Bruintjes et al. further suggested that the so-called dermocartilaginous ligament may represent a surgical dissection artifact rather than a true anatomical entity.

In 2003, Huizing analyzed the persistent confusion surrounding nasal ligament terminology. He argued that terms such as soft triangle, weak triangle, Pitanguy ligament, and scroll ligament lack consistent anatomical and embryological support, advocating instead for descriptive terminology based on the actual morphology of the SMAS and perichondrial planes. Together with the work of Zhai et al. [4], these studies demonstrated that structures traditionally described as the interdomal, intercrural, and septocrural ligaments do not exist as discrete histologic entities because the connective fibers run parallel to the cartilages rather than forming true transverse ligamentous connections [7].

Similarly, Zhai et al. demonstrated through anatomical dissections and histologic studies that a true interdomal ligament does not exist. Instead, they identified a continuous fibro-adipose tissue plane extending between the middle crura [4].

More recently, Popko et al. revisited the anatomy of nasal tip support and confirmed that many previously described ligaments lack distinct histologic boundaries, representing instead connective tissue condensations or extensions of the nasal SMAS [9]. These findings reinforced the concept that nasal support is provided by a continuous fascial network linking the skin, the SMAS, and the osseocartilaginous framework rather than by isolated ligamentous structures.

In 2025, Ku et al. described three consistently identifiable nasal retaining ligaments in Asian cadavers: superior, middle, and inferior. The superior retaining ligament originated near the medial canthus and inserted into the nasal bone, the middle ligament was located in the K-area, and the inferior retaining ligament, also referred to as the vertical scroll ligament, connected the SMAS to the junction between the upper and lower lateral cartilages [5]. These fibromuscular structures function as vertical anchoring points between the osseocartilaginous framework, the SMAS, and the skin. The authors suggested that controlled release of these retaining ligaments may facilitate nasal lengthening and projection during structural rhinoplasty, particularly in patients with thick skin, whereas their preservation may improve skin redraping and postoperative definition [5].

Also in 2025, Neves et al. proposed the Fusion Sling concept, an embryologically based reinterpretation of nasal support anatomy. According to this model, fusion of the medial and lateral nasal processes creates a continuous suspensory network connecting the upper and lower lateral cartilages while dividing the nasal tip into three anatomical compartments: the supratip, intercrural region, and membranous septum [6]. Within this framework, the SOON was identified as the most superficial muscular layer of the nasal SMAS, consisting of orbicularis oris nasalis muscle fibers and connective tissue located within the infratip and columellar regions. Neves et al. proposed that this structure occupies the intercrural compartment and contributes to infratip support and mobility without extending superiorly beyond the domes [6].

This concept also supports the interpretation that the so-called Pitanguy ligament is not an isolated ligament but rather a fascial continuation of the nasal SMAS. Consequently, several authors have suggested that the term Pitanguy flap more accurately reflects its anatomical nature than Pitanguy ligament [6-10].

From a surgical standpoint, these findings do not necessarily imply that all fibrous condensations should be preserved. As emphasized by Rohrich et al., the objective of structural rhinoplasty is often to intentionally modify the native anatomy by releasing selected retaining structures, reconstructing the nasal framework, and subsequently eliminating dead space through controlled soft-tissue repositioning [1].

The SOON Flap Stitch technique is directly based on this contemporary anatomical understanding. Rather than routinely resecting the soft tissues of the intercrural compartment, the technique preserves the SOON muscle as a vascularized flap and repositions it over the reconstructed columellar framework. This approach is intended to restore soft-tissue continuity, occupy the intercrural space, and preserve columellar and infratip soft-tissue volume while facilitating redraping of the superficial nasal SMAS.

Study objective

This technical report describes the SOON Flap Stitch technique and its clinical application in 226 consecutive primary open rhinoplasty procedures involving patients with thin, intermediate, and thick skin. All procedures were performed by Dr. Alan Burgos in Mazatlán, Sinaloa, Mexico, between February 2022 and November 2024. Postoperative follow-up ranged from eight months to three years. This clinical experience is presented to illustrate the application and reproducibility of the technique across different skin thicknesses rather than as a comparative or outcomes-based cohort study.

## Technical report

Surgical technique

An open rhinoplasty approach was performed through an inverted-V transcolumellar incision. After elevation of the skin-soft-tissue envelope, the superficial layer of the nasal SMAS, composed of fibers of the SOON muscle at the infratip region, was carefully identified and preserved.

At the completion of the procedure, the preserved SOON flap was repositioned over the columella and secured to the superficial plane using 5-0 polypropylene (Prolene) suture. This maneuver creates an autologous soft-tissue augmentation effect by restoring columellar volume, enhancing infratip definition, providing additional structural support, and reducing dead space.

The complete surgical technique is demonstrated in Video 1.

**Video 1:** Step-by-step demonstration of the SOON Flap Stitch technique during open structural rhinoplasty. The video illustrates the identification and preservation of the SOON flap, followed by its repositioning and fixation to the columella during closure to restore columellar volume, improve infratip support, and reduce dead space. SOON: superficial orbicularis oris nasalis

Clinical outcomes

Cases in which preservation and incorporation of the SOON flap were feasible showed favorable qualitative observations in soft-tissue redraping and the maintenance of columellar and infratip contour. The technique was also observed to provide soft-tissue coverage of the intercrural space following the reconstruction of the underlying framework. These observations were based on clinical assessment and two-dimensional photographic follow-up and were not evaluated using comparative, volumetric, or validated patient-reported outcome measures. No major technique-related complications were observed during the available follow-up period (Figures 1-3).

**Figure 1:**
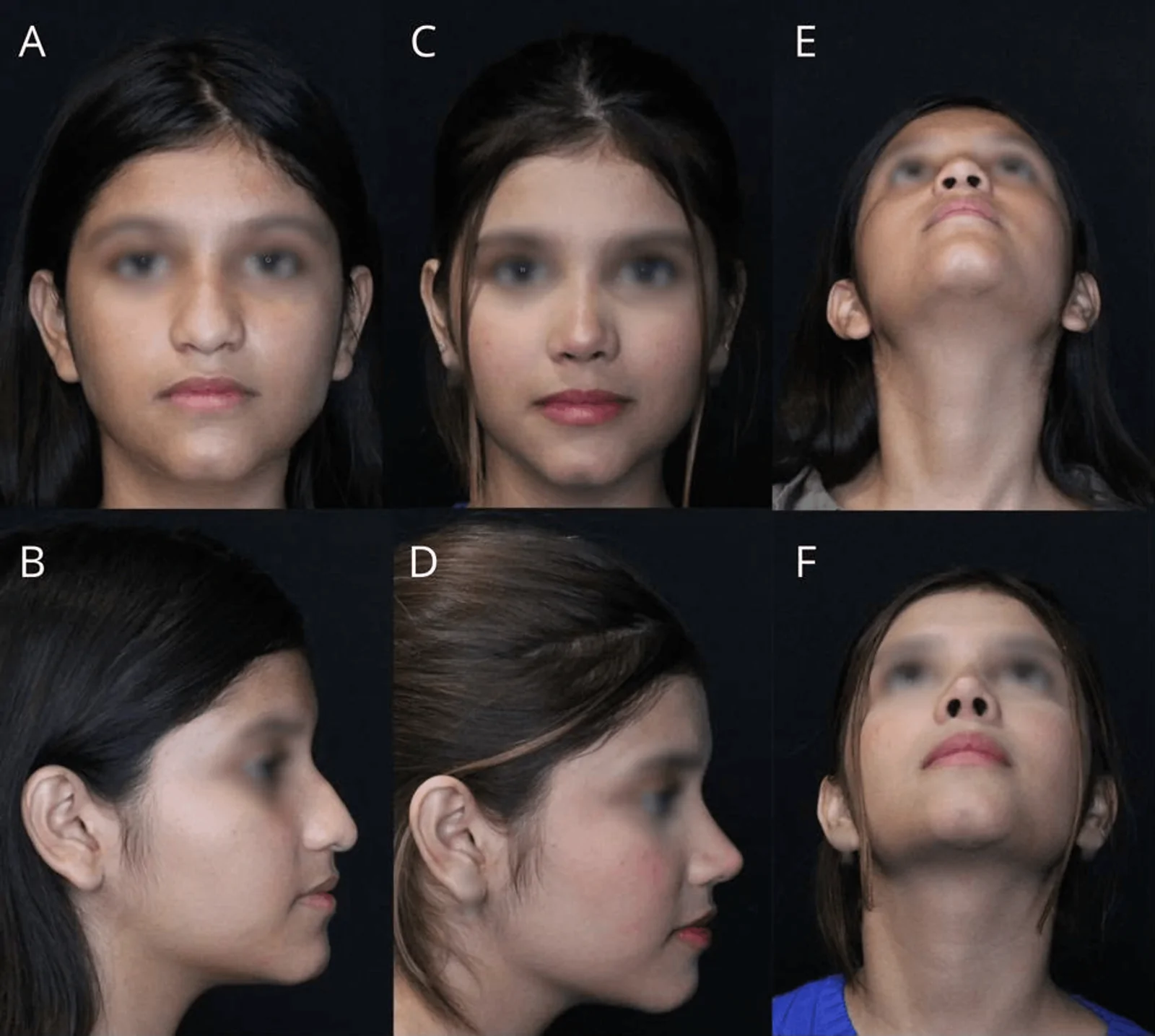
Patient with intermediate-thickness skin. (A) Preoperative frontal view. (B) Preoperative right lateral view. (C) Postoperative frontal view. (D) Postoperative right lateral view. (E) Preoperative basal (worm's-eye) view. (F) Postoperative basal (worm's-eye) view. The additional basal views allow direct visualization of the columellar and infratip contours and provide complementary assessment of the infratip-columellar relationship.

**Figure 2:**
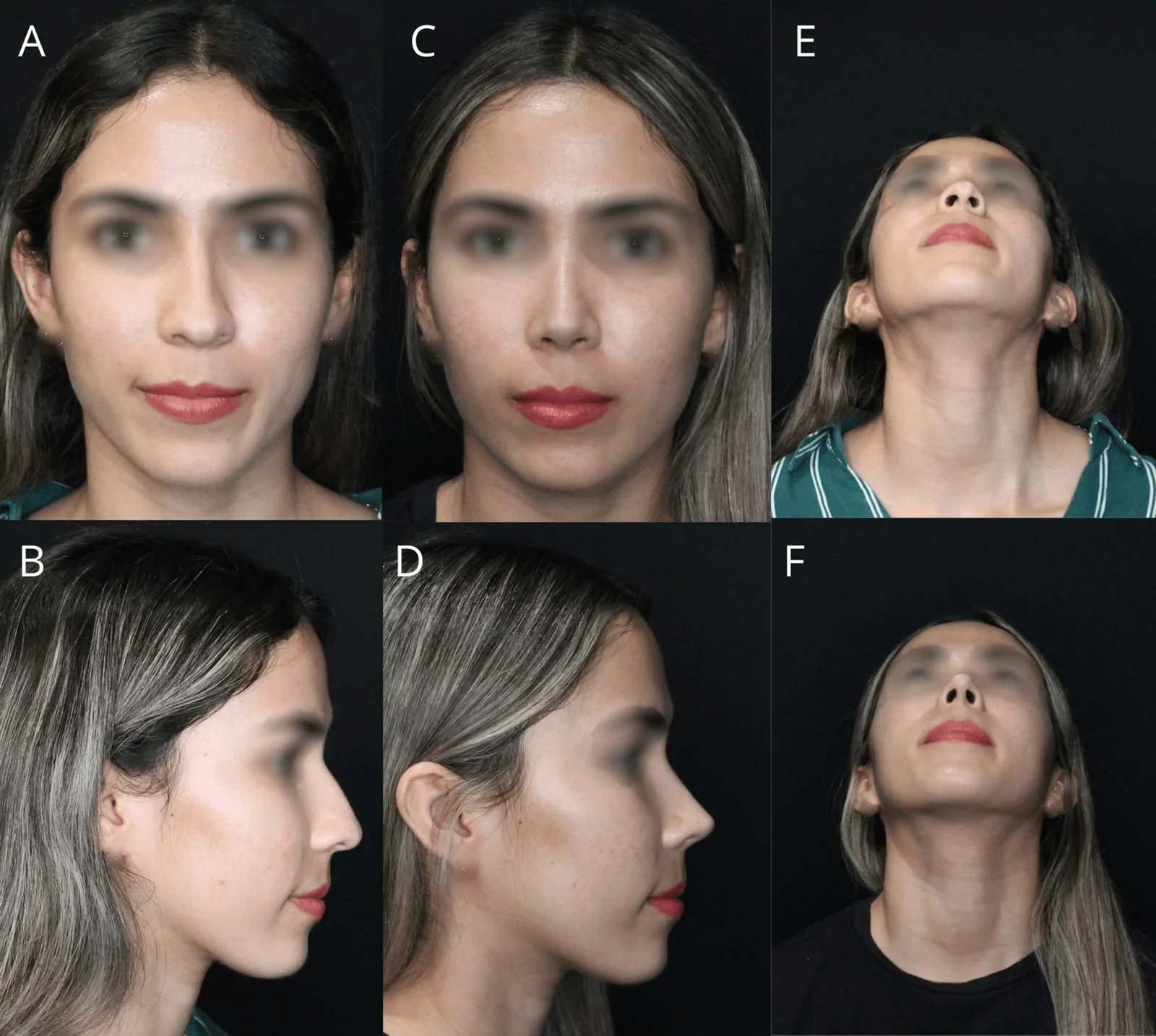
Patient with thin skin. (A) Preoperative frontal view. (B) Preoperative right lateral view. (C) Postoperative frontal view. (D) Postoperative right lateral view. (E) Preoperative basal (worm's-eye) view. (F) Postoperative basal (worm's-eye) view. The additional basal views allow direct visualization of the columellar and infratip contours and provide complementary assessment of the infratip-columellar relationship.

**Figure 3:**
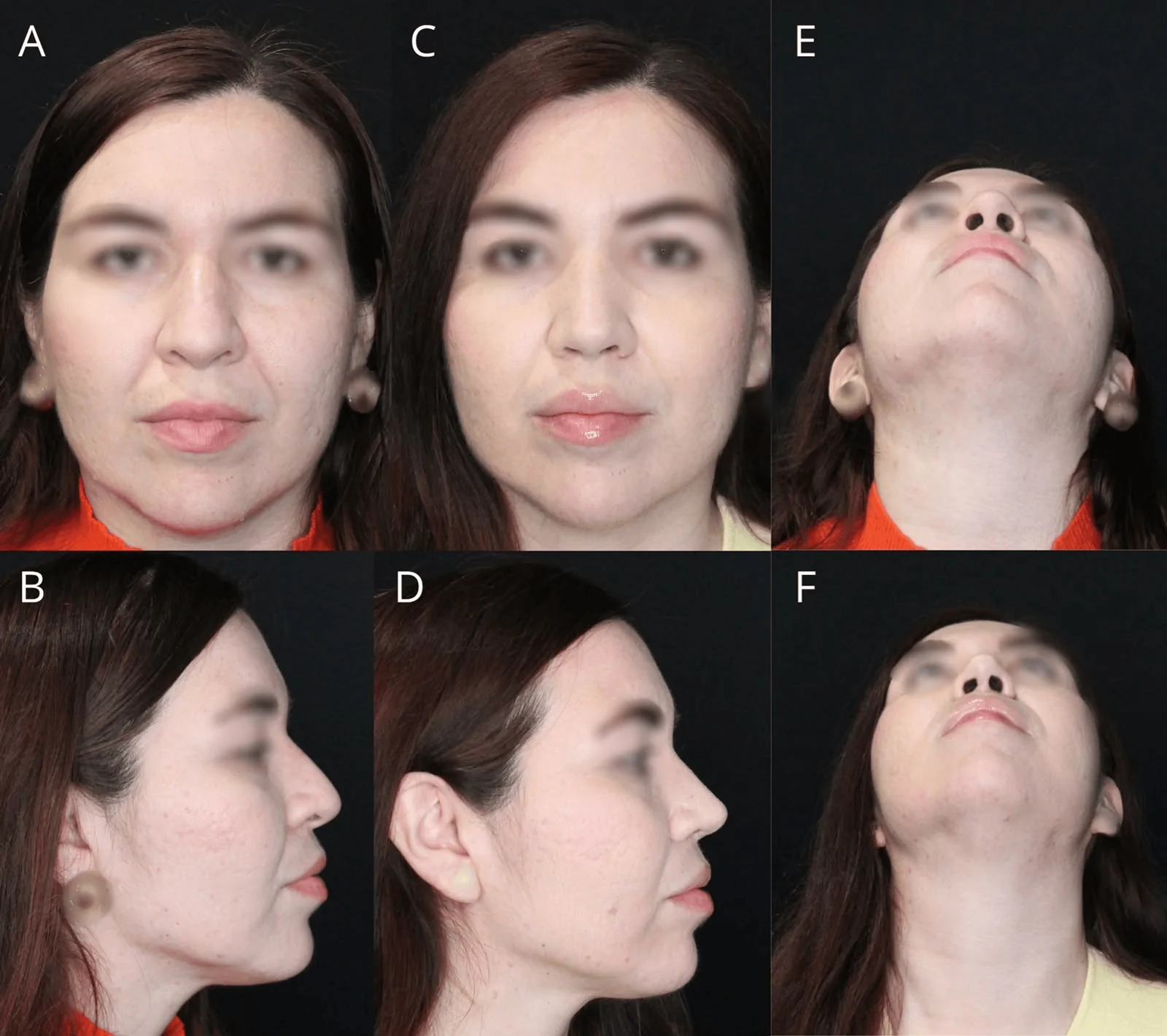
Patient with thick skin. (A) Preoperative frontal view. (B) Preoperative right lateral view. (C) Postoperative frontal view. (D) Postoperative right lateral view. (E) Preoperative basal (worm's-eye) view. (F) Postoperative basal (worm's-eye) view. The additional basal views allow direct visualization of the columellar and infratip contours and provide complementary assessment of the infratip-columellar relationship.

## Discussion

The management of dead space is a fundamental component of structural rhinoplasty. Rohrich et al. emphasized that controlling the space between the skin-soft-tissue envelope, including the SMAS, and the underlying osseocartilaginous framework is essential to minimize fibrosis, preserve soft-tissue definition, and reduce postoperative aesthetic deformities, particularly in the infratip region [1]. The SOON Flap Stitch technique directly addresses this principle by providing biological closure of the intercrural dead space using vascularized autologous tissue.

The nasal retaining ligaments described by Ku et al. and the nasal suspensory network proposed by Neves et al. demonstrate that the nasal soft-tissue envelope is stabilized by a complex arrangement of vertical and horizontal fibromuscular condensations [5,6]. However, as previously reported by Bruintjes et al. and later confirmed by Popko et al., these anatomical structures are not newly identified but have been recognized for several decades [2,9]. Neves et al. integrated these embryological concepts into the Fusion Sling model, in which the SOON forms part of the nasal support system [6]. Likewise, Huizing emphasized that accurate anatomical nomenclature is essential for standardizing terminology and facilitating communication of surgical techniques [7].

As a technical report, this article is intended primarily to describe the anatomical rationale, surgical steps, and clinical application of the SOON Flap Stitch rather than to establish its independent efficacy. The clinical observations presented here were not obtained from a comparative study and were not assessed using three-dimensional volumetric analysis or validated patient-reported outcome measures. Therefore, the specific contribution of the SOON flap cannot be isolated from other components of framework reconstruction, grafting, and suturing performed during rhinoplasty. Future prospective comparative studies incorporating standardized three-dimensional analysis and validated outcome instruments, such as the Rhinoplasty Outcome Evaluation (ROE) or Standardized Cosmesis and Health Nasal Outcomes Survey (SCHNOS), may help objectively evaluate the contribution of this technique to postoperative soft-tissue contour and patient-reported outcomes.

## Conclusions

The SOON Flap Stitch represents an anatomically based refinement in open structural rhinoplasty designed to preserve and reposition the superficial nasal SMAS over the reconstructed columellar framework. By incorporating vascularized autologous tissue into the intercrural space, the technique aims to maintain soft-tissue continuity, minimize dead space, and preserve columellar and infratip contour. The technique was feasible across different skin thicknesses and was not associated with major technique-related complications during the available follow-up. These observations are qualitative and should not be interpreted as evidence of independent efficacy. Further prospective comparative studies incorporating objective outcome measures are warranted. Based on the anatomical principles presented in this technical report, preservation and incorporation of the SOON flap and tissues of the intercrural compartment may be considered whenever anatomically feasible. The SOON Flap Stitch may therefore represent a useful adjunct to contemporary SMAS preservation and soft-tissue envelope management strategies in open rhinoplasty.
